# Inosine Enhances Axon Sprouting and Motor Recovery after Spinal Cord Injury

**DOI:** 10.1371/journal.pone.0081948

**Published:** 2013-12-02

**Authors:** Daniel Kim, Laila Zai, Peng Liang, Colleen Schaffling, David Ahlborn, Larry I. Benowitz

**Affiliations:** 1 Laboratories for Neuroscience Research in Neurosurgery, Children’s Hospital, Boston, Massachusetts, United States of America; 2 F.M. Kirby Neurobiology Center, Children’s Hospital, Boston, Massachusetts, United States of America; 3 Surgery Department, Harvard Medical School, Boston, Massachusetts, United States of America; 4 Ophthalmology Department, Harvard Medical School, Boston, Massachusetts, United States of America; 5 Program in Neuroscience, Harvard Medical School, Boston, Massachusetts, United States of America; Hertie Institute for Clinical Brain Research, University of Tuebingen., Germany

## Abstract

Although corticospinal tract axons cannot regenerate long distances after spinal cord injury, they are able to sprout collateral branches rostral to an injury site that can help form compensatory circuits in cases of incomplete lesions. We show here that inosine enhances the formation of compensatory circuits after a dorsal hemisection of the thoracic spinal cord in mature rats and improves coordinated limb use. Inosine is a naturally occurring metabolite of adenosine that crosses the cell membrane and, in neurons, activates Mst3b, a protein kinase that is part of a signal transduction pathway that regulates axon outgrowth. Compared to saline-treated controls, rats with dorsal hemisections that were treated with inosine showed three times as many synaptic contacts between corticospinal tract collaterals and long propriospinal interneurons that project from the cervical cord to the lumbar level. Inosine-treated rats also showed stronger serotonergic reinnervation of the lumbar cord than saline-treated controls, and performed well above controls in both open-field testing and a horizontal ladder rung-walking test. Inosine was equally effective whether delivered intracranially or intravenously, and has been shown to be safe for other indications in humans. Thus, inosine might be a useful therapeutic for improving outcome after spinal cord injury.

## Introduction

Spinal cord injury (SCI) can result in devastating, irreversible losses of sensory, motor, and/or autonomic function due to the interruption of connections between higher brain centers and segments of the spinal cord below the level of injury. Multiple factors normally prevent these pathways from regenerating, and in animal models of SCI, significant improvements have been achieved by counteracting inhibitory molecules associated with myelin and the glial scar, inserting stem cell bridges and/or biopolymer scaffolds, providing growth factors or cAMP analogs, altering gene expression, and/or increasing physiological activity [1-8]. However, this research has not yet led to novel therapeutic interventions and some of these approaches carry potential risks. 

Although long-distance regeneration remains a major challenge, the corticospinal tract (CST) and other major pathways can form compensatory circuits after incomplete lesions. Transection of the CST at the thoracic level causes severed axons to sprout collateral branches at the cervical level that synapse onto long propriospinal interneurons (LPSNs), forming “detour circuits” that restore some cortical control to the hindlimbs [9-11]. Undamaged CST axons can also form collateral branches that contribute to recovery [12]. Thus, agents that promote sprouting may help improve outcome after partial lesions of the spinal cord. 

One such agent is inosine, a naturally occurring derivative of adenosine. Inosine diffuses into neurons and activates Mst3b, a protein kinase that is part of a signal transduction pathway that regulates axon growth [13,14]. Inosine induces outgrowth in several types of neurons in culture and, when infused into the brain after unilateral cortical injury, enables CST axons that arise from the undamaged hemisphere to extend collateral branches into the denervated side of the spinal cord [13,15-21]. 

The present study investigated whether inosine can augment the formation of detour circuits and improve functional outcome after transecting the CST and other pathways in the dorsal spinal cord of mature rats. We report that inosine more than tripled the formation of CST synapses onto LPSNs in the cervical cord after a dorsal hemisection at the thoracic level, increased raphespinal sprouting, and markedly improved fine motor coordination as well as general locomotion. Inosine was equally effective whether given intravenously or intracerebrally. Because of its safe history of use in humans for other indications, inosine is an attractive candidate for clinical trials in SCI patients.

## Materials and Methods

### Ethics Statement

All procedures were performed in accordance with NIH guidelines and were approved by Boston Children’s Hospital’s Institutional Animal Care and Use Committee (IACUC, protocol 13-04-2373R). All animals used in the study were anesthetized by either a Ketaset/dexdormitor cocktail (dosages below) or isoflurane and were maintained in a deep state of anesthesia throughout all surgical procedures. Animals were euthanized with an overdosed by isoflurane prior to transcardial perfusion (details below). All efforts were made to minimize suffering. 

### Spinal Cord Surgery

Adult male Sprague Dawley rats (250-300 g; Charles River Laboratories) were maintained on a 12 h light/dark cycle. Experimental and control cases were generated on a random basis, and the subsequent analyses of behavior and anatomy were blinded. Animals included in the study were generated over a course of 2 years, with no more than two surgeries on a single day. Rats were anesthetized with Ketaset (ketamine, 75 mg/kg, s.c.; Fort Dodge) and dexdormitor (dexmedetomidine, 0.25 mg/kg, s.c.; Pfizer), and a skin incision was made at the T8 level. Muscles were cut in layers along the midline and retracted under a surgical microscope. A laminectomy was performed at the T8 level. Lidocaine-HCl (10 mg/ml; Hospira) was applied topically to the spinal cord to minimize pain and sensation during surgery. The dura mater was opened and removed using #3 jewelers’ forceps and a #11 scalpel blade. The dorsal half of the spinal cord was transected using a #11 scalpel blade to a depth of 1.1 mm. Transection of the dorsal CST was confirmed visually and subsequently verified histologically. Gelfoam was packed into the injury site and muscles were closed in layers using 3-0 silk sutures. Sham-operated animals served as normal controls and underwent all procedures up to the point of the dorsal hemisection and pump placement. 

### Osmotic Minipumps

Immediately after spinal cord surgery, rats were fitted with Alzet osmotic minipumps to deliver saline or inosine either intracerebroventricularly (*i.c.v.*) or intravenously (*i.v.*). Minipumps were prepared the day before for priming per the manufacturer’s instructions (Durect). Animals receiving *i.c.v.* treatment had a borehole drilled over the lateral ventricle of the right hemisphere (coordinates: 0.08 cm A/P, 0.14 cm M/L relative to bregma) and an Alzet brain infusion needle (Brain Infusion Kit 2; Durect) was inserted to a depth of 5.0 mm and secured using a silicon-based glue. The brain infusion kit was attached to an osmotic pump (Alzet 2002; Durect) that was placed subcutaneously behind the shoulder blades. Animals in all experiments were assigned on a random basis to receive either saline (0.9%, Baxter) or inosine (50 mM in saline, Sigma-Aldrich) at a flow rate of 0.5 µl/hour (~ 0.5 mg/kg body weight/day). Pumps were replaced biweekly. 

For intravenous (*i.v.*) delivery, rats were purchased from Charles River Laboratories with pre-fitted jugular vein catheters. Immediately after surgery, rats were fitted with Alzet osmotic pumps (2ML1, Durect) attached to the catheters to deliver saline or inosine (260 mM, ~ 70 mg/day/kg body weight). In a subsequent dose-response study, rats also received *i.v.* inosine at either 30 or 100 mM (8 - 27 mg/day/kg body weight). Pumps were changed weekly. To ensure that catheters remained functional, they were flushed weekly during the pump change with heparin lock flush (100 USP Units/ml, APP Pharmaceuticals). Inosine solubility increases with elevated pH, and we adjusted the pH of both the inosine and vehicle (saline) solutions to 9.2 to allow solubilization of the highest concentration used. Intravenous delivery of inosine elevates cerebrospinal fluid concentrations within 45 minutes of beginning infusion (Hurtt et al., U.S. Patent 20090221521). Following pump placement, the skin was closed with 3-0 silk sutures and rats were allowed to recover on a warming pad before being returned to the animal facility. All animals were given Buprenex (0.05 mg/kg, s.c.; buprenorphine; Bedford Laboratories) every 8-12 hours for 72 hr and the antibiotic Enrofloxacin (2.5 mg/kg, s.c., Bayer) daily for one week. Bladders were expressed manually twice daily until voluntary control was restored. Rats received subcutaneous boluses of lactated Ringer’s solution twice daily for 2 weeks to maintain hydration. 

### Behavioral Testing

Rats were tested for general locomotion using the Basso, Beattie, Bresnahan (BBB) scale [22] and for skilled use of the hindlimbs using the horizontal ladder rung walking test [23]. The BBB was evaluated at 24 hours and 7 days after surgery and then weekly over a 4-week period. Two observers were trained to assess hindlimb function according to standard criteria [22] and scored each behavioral exam independently. The number of rats tested in Study I was: saline (combined *i.v. and i.c.v.*), n = 14; inosine *i.c.v.*, n = 10; inosine *i.v.*, n = 8. In Study II: saline *i.v.*, n = 14; inosine *i.v.*, n = 20. The same rats were evaluated for skilled use of the hindlimbs using a horizontal ladder rung walking test the week after spinal cord surgery and every week after that over the 4-week period (n values same as above). This task assesses sensorimotor coordination and requires descending cortical control [23]. Animals were placed on a horizontal ladder 14 cm wide and 100 cm long suspended 30 cm above the surface. Rungs were spaced irregularly at 2 - 5 cm intervals to prevent rats from learning a fixed pattern of stepping. Performance was video-recorded and scored later (successful steps/total steps) by lab members blind to animals’ treatments. Unsuccessful steps are as defined by Metz and Whishaw [23], where a hindlimb either misses a rung completely or initially makes contact with a rung but slips during weight-bearing. 

### Anatomical Tracing

Twenty-eight days after SCI, animals received bilateral injections of the anterograde tracer biotinylated dextran amine (BDA, 10,000 MW, 10% w/v solution in sterile saline; Invitrogen) into 9 sites within the hindlimb motor area under stereotaxic guidance. In the first study, BDA placement was guided by the boundaries of the hindlimb area shown in a standard rat brain atlas [24]. We subsequently discovered, however, that these injections infringed upon the forelimb area, causing considerable labeling of CST axons that normally project to the cervical spinal cord. This labeling made it difficult to assess the sprouting of hindlimb CST fibers after SCI, and we therefore modified the injection coordinates based on an electrophysiological mapping study of the motor cortex [25] (new coordinates in cm with respect to bregma: 0.05 A/P, ± 0.10 M/L; 0.00 A/P, ± 0.10 M/L; 0.00 A/P, 0.0 M/L; -0.05 A/P, ± 0.10 M/L; -0.05 A/P, ± 0.15 M/L; -0.10 A/P, ± 0.10 M/L; -0.10 A/P, ± 0.15 M/L). BDA injections (Nanoject II, Drummond Scientific) were placed at 3 different depths (2.0 mm, 1.0 mm, 0.5 mm, 1 min each) for each injection site. Retrograde labeling of long propriospinal interneurons was carried out by injecting 1 µl of Cholera Toxin Subunit B (CTB; 1%; List Biological) into each side of the lumbar spinal cord (level L2 – L3) using a glass micropipette and Nanoject II (200-300 µm lateral to midline; ~ 400 µm depth). Micropipettes remained in place for 1 min after injections. The skin and muscles were then sutured closed and rats recovered on a warming pad before returning to their cages. Two weeks later, rats were euthanized with an overdose of anesthesia and perfused transcardially with 0.9% saline followed by 4% paraformaldehyde (PFA). The brain and spinal cord were post-fixed in 4% PFA overnight (4°C) and cryoprotected in sucrose (10% for 24 h, then 30% for 24 h). A 2 cm segment of the thoracic spinal cord centered around the lesion site was embedded in gelatin-albumin (Type-A; Sigma-Aldrich) and cut at 40 µm in the parasagittal plane using a Vibratome. The brain and portions of the cervical and lumbar spinal cord were embedded in Tissue-Tek (Sakura Finetek USA) and frozen (–20°C) for cryostat sectioning. Although rats from the first study were not analyzed for axon sprouting, they were analyzed for behavior, lesion depth, and serotonergic reinnervation, which are unaffected by the tracer injections. 

### Visualization of BDA injections

Free-floating sections through the forebrain (coronal plane, 40 µm thick) were reacted with avidin-biotin complex conjugated to horseradish peroxidase (Vectastain ABC Kit, Vector) followed by a DAB-peroxidase substrate kit (Vector). Sections were mounted onto slides (Superfrost Plus; VWR), dried, rehydrated through graded ethanol solutions, stained with Cresyl Fast Violet (Certified; Ted Pella, Inc.), dehydrated through serial ethanol solutions and xylene, and covered using Permount (Fisher). BDA placement was confirmed anatomically in each rat by reference to a standard brain atlas [24]. The observation that very few CST fibers were labeled in the cervical enlargement of sham-operated controls provides further evidence that our BDA injections did not infringe into the forelimb motor area.

### Lesion Depth and Area

The cross-sectional area of the lesion and width of the remaining tissue bridge were measured using NIH ImageJ software (Wayne Rasband, NIH, Bethesda, MD), averaging the results from several sections close to the midline. To further verify that we had transected the CST, free-floating coronal sections at the cervical and lumbar levels (40 µm) were stained to visualize BDA-positive axons as above. Rats were excluded from the study if (i) spared tissue at the center of the lesion was either ≤ 15% or ≥ 50% the width of the spinal cord (measured in adjacent uninjured areas); or if (ii) BDA-positive fibers were seen in the dorsal CST in the lumbar region. These criteria excluded rats with lesions that spared fibers in the principal (dorsomedial) CST or that were too large to allow functional recovery (see below). Lesion area is presented as the percentage of tissue that was lost or necrotic in an area spanning 4 mm on either side of the lesion epicenter. 

### CST Sprouting

Coronal sections through levels C3 – C5 were stained to visualize BDA-positive axons as above. We identified the midline using the central canal and dorsal median fissure as landmarks, then quantified the number of BDA-positive axon profiles ≥ 50 μm in length within the gray matter at the edge of the dorsal CST, at the midline (M), and at two fixed distances from the midline labeled Zones I and II (see below). Zone I was defined by lines parallel to the midline passing through the lateral edges of the dorsal CST on either side, whereas Zone II included virtual lines at twice this distance from the midline. Results were averaged over 12-20 sections and were normalized by the total number of labeled axons in the dorsal CST for each case. This latter number was established by staining 10 coronal sections through the cervical enlargement with fluorescently tagged strepavidin-biotin complex (Strepaviden Alexa Fluor 647, Invitrogen) and capturing digital images through 3 µm optical sections (Ultraview Vox Spinning Disk confocal microscope). Axons, which appear as dots in the coronal plane, were quantified using Stereo Investigator (MBF Bioscience) and averaged across the 10 sections to determine the number of labeled axons in the CST for each case. 

### CST contacts onto LPSNs

Free-floating sections through cervical levels C3-C5 were blocked with a solution containing 3% bovine serum albumin (BSA) and 10% normal goat serum (NGS) in PBS with 0.5% Triton X-100, then incubated in a rabbit anti-CTB antibody (1:10,000, Genway) in a similar buffer but with 5% NGS overnight at 4°C. Sections were rinsed and incubated with goat anti-rabbit IgG conjugated to Alexa Fluor 594, 1:500, and Strepavidin conjugated to Alexa Fluor 647, 1:1000 (Invitrogen). Digital images of all CTB-positive cells (typically 4-7 per section, avg. 45/case) were captured in 10-12 sections, and the number of BDA-labeled boutons, defined as swellings at least twice the thickness of the axon [26] falling on or within 20 µm of a CTB-labeled soma or dendrite, were quantified using the Stereo Investigator program. All CTB-labeled somata were counted and results are presented as the number of BDA-positive contacts onto CTB-positive LPSNs per section normalized by the number of BDA-positive axons in the CST and averaged over the experimental group.

### Synaptophysin-positive contacts

A separate set of free-floating sections through the cervical enlargement was rinsed in PBS, transferred to a preheated solution of 10 mM sodium citrate (pH 8.5, 80° C, 30 min), then to 3% H_2_O_2_ in methanol (10 min), blocked with 5% NGS in PBS, and incubated overnight with a monoclonal anti-synaptophysin antibody (1:200, Millipore) in PBS containing 3% Triton X-100 at 4°C. Sections were rinsed in PBS and incubated in an Alexa Fluor 488-conjugated antibody to mouse IgG (made in goat: Invitrogen) in 0.3% Triton X-100 in PBS and for 2 h, RT. Sections were rinsed and stained for BDA in presynaptic axons and CTB in long propriospinal interneurons as above. Sections were then mounted and covered. Stacked images were collected in the vicinity of all CTB-positive cell bodies (Ultraview Vox confocal) and transferred to the Stereo Investigator software. This enabled us to quantify (i) total BDA-positive bouton-like swellings, (ii) total BDA+ boutons that were double-stained for synaptophysin, and (iii) Synaptophysin-positive, BDA-positive terminals that fell on or within 20 µm of CTB-positive long propriospinal interneurons. Results were averaged over 10-12 sections and normalized by the total number of BDA-positive axons in the CST as evaluated above.

### 5-HT quantitation

Free-floating sections through the lumbar enlargement (L1-3) were rinsed in high-salt buffer (HSB), blocked with 5% NGS in HSB containing 0.3% Triton X-100, incubated with a rabbit anti-5HT antibody (1:40,000, Immunostar), rinsed thoroughly, and incubated with an Alexa Fluor 488-conjugated antibody to rabbit IgG made in goat (Invitrogen). A stacked image of the ventral horn was captured (Ultraview Vox Spinning Disk confocal microscope), and the integrated density of 5HT immunoreactivity was quantified using ImageJ software. Results were averaged over 10-12 sections per case and then over the experimental group.

### Statistics

Results are presented as group means ± SEM based on ≥ 6 rats per group. Statistical analyses used Mann-Whitney rank-sum tests and, where appropriate, one-way and two-way ANOVA. Scatter plots and Pearson’s product-moment correlation coefficients were used to evaluate the relationship between behavioral scores and lesion depth. Reported *P* values are all two-tailed.

## Results

### Improvement of hindlimb function after inosine treatment

Three-to-four weeks after a dorsal hemisection of the thoracic spinal cord, rats treated with inosine performed markedly better than saline-treated controls on tests of general locomotion and fine-motor coordination. Inosine was equally effective whether administered intracranially or intravenously. The behavioral effects of inosine were seen in two independent studies.

#### Study I: Intravenous vs. intracerebroventricular delivery

In the first of two studies, we compared the efficacy of inosine delivered by direct infusion into the lateral ventricle (intracerebroventricularly, *i.c.v.*, n = 5) vs. intravenously (i.v., n = 7). *i.c.v.* delivery of inosine has been shown to enhance CST sprouting and to improve skilled use of an affected paw after stroke or traumatic brain injury [18,20,21,27]. Clinically, however, *i.c.v.* delivery is undesirable due to the risk of intracranial infection, and we therefore investigated whether *i.v.* delivery would be equally effective. Vehicle-treated controls in the present study performed similarly regardless of whether saline was delivered *i.c.v.* or i.v., and we therefore pooled these animals to increase statistical reliability (final BBB score for i.c.v. saline = 13.9 ± 1.3, for i.v. saline = 13.1 ± 1.1; average = 13.5 ± 0.9; ladder rung walking test score for i.c.v. saline at 4 weeks = 19.9 ± 14.1%, for i.v. saline = 21.8 ± 8.6%; avg. = 21%). 

**Figure 1:**
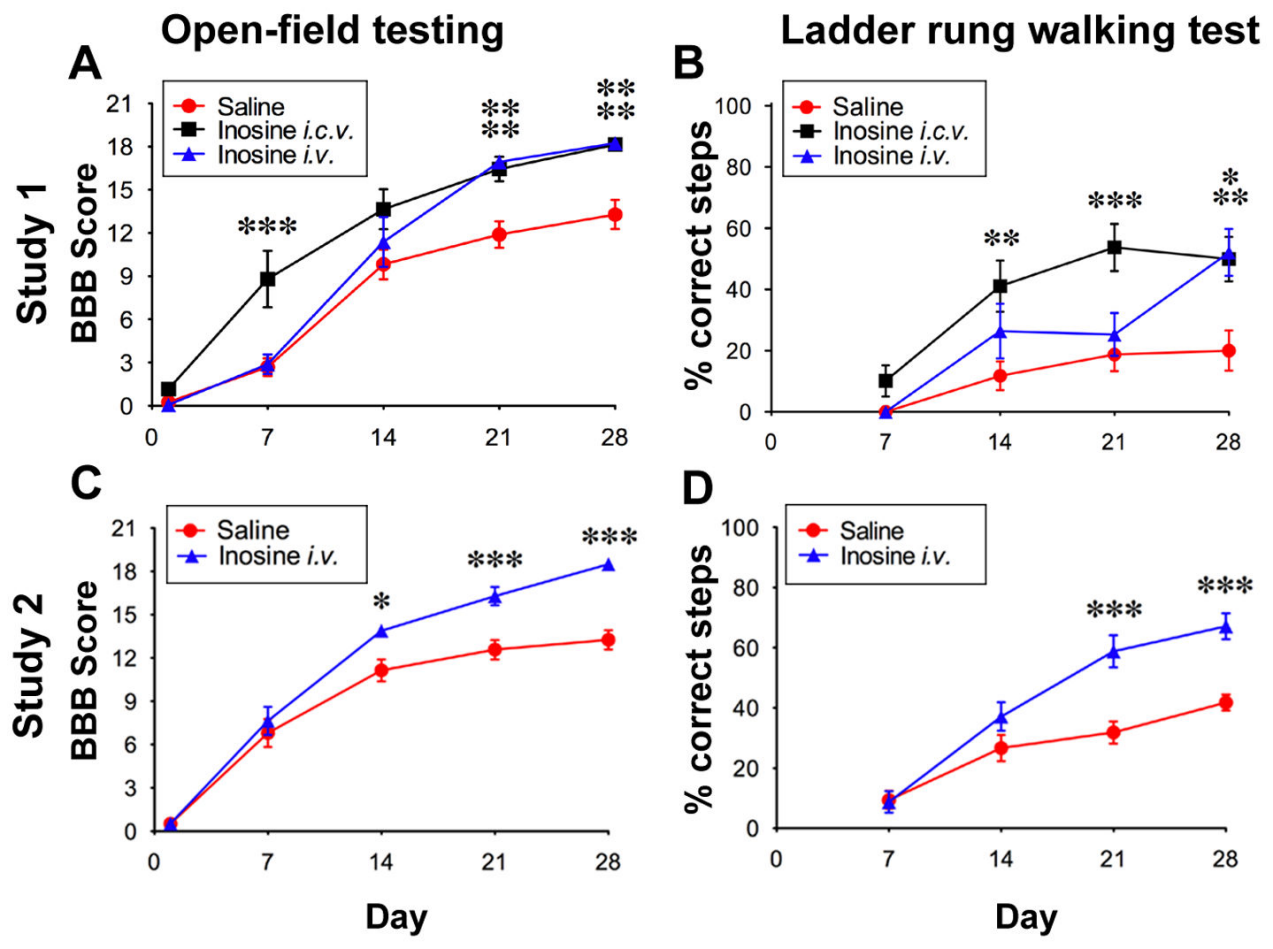
Inosine improves open-field behavior and skilled use of hindlimbs. Behavioral studies were carried out in 2 separate cohorts of rats. General locomotor function was evaluated using the Basso-Beattie-Bresnahan (BBB) scale, whereas fine motor coordination was tested using the ladder rung-walking test, scoring the percentage of steps in which either hindlimb was placed on a rung without error. **A**, **B**. Study 1. Inosine was delivered either i.c.v. (black) or i.v. (blue); saline-treated controls are shown in *red*. Note the superior performance of animals treated with either i.c.v. or i.v. inosine on the BBB (**A**) and the ladder rung walking test (**B**). **C**, **D**, Study 2. Inosine and saline were delivered i.v. Inosine-treated rats again performed better than saline-treated controls on both the BBB (**C**) and ladder-rung walking test (**D**) at weeks 3 and 4. *P < 0.05, **P < 0.01, ***P < 0.001. Data in all figures represent group means ± SEM (Two-way ANOVA, Bonferroni *a* *priori* test).

All groups were severely impaired in open-field behavior one day after surgery (BBB score ≤ 3: Figure 1A), possibly reflecting spinal shock and secondary injury. Inosine-treated rats (*i.c.v.*, n = 13; *i.v.*, n = 6) began to outperform controls by two weeks and maintained this superiority for the remainder of the study, averaging 4 points higher on the BBB scale at 4 weeks (Figure 1A: F = 21.35, P < 0.01). Rats performed similarly after 4 weeks regardless of whether inosine was delivered *i.v.* or *i.c.v.* (Figure 1A). Rats receiving inosine *i.c.v.* appeared to recover somewhat faster than those receiving *i.v.* inosine, possibly due to the greater bioavailability in the former case. However, in our second study (see below), the rate of recovery in the *i.v.* inosine group was comparable to that of the i.c.v. inosine group in Study I. The difference in performance between inosine- and saline-treated rats after 4 weeks translates into a greater consistency of forelimb-hindlimb coordination, plantar stepping, toe clearance, and positioning of the hindlimb parallel to the direction of movement. 

Unlike open-field behavior, which is largely independent of the CST [28,29], the ladder rung-walking test requires this pathway [23]. Inosine-treated rats outperformed controls on this test from the second week on (P < 0.01, F = 14.56), and by 4 weeks, they were able to place their hindlimbs correctly on the ladder rungs ≥ 50% of the time (Figure 1B). As with open-field behavior, the final scores of inosine-treated rats on the ladder rung test were nearly identical regardless of whether inosine was delivered *i.v.* or *i.c.v*. 

#### Study II: Confirmation of i.v. inosine effects

After completing the first study, we discovered that our BDA injections infringed upon the forelimb motor area. This labeled many axons that normally project to the cervical enlargement and made it difficult to detect changes in collaterals that arise from injured hindlimb axons. We therefore carried out a second study in which we restricted BDA injections to the hindlimb area as defined by electrophysiological mapping [25]. As noted below, the new injections avoided labeling the forelimb motor area, as judged by the scarcity of BDA-positive axons in the cervical gray matter of control rats without SCI. Based on the results of the first study and the greater clinical relevance of i.v. vs. i.c.v. delivery, rats in the second study only received *i.v.* infusions. Because of the problem with BDA placement in Study I, all CST sprouting results are based on rats in Study II*.*

Rats receiving *i.v.* inosine (n = 20) again outperformed saline-treated controls (n = 14) by a wide margin on the BBB scale, scoring 5 points higher on average at the end of the 4-week testing period (Figure 1C: F = 354, P < 0.001). Inosine-treated rats in the second study were also superior to saline-treated controls in the ladder rung-walking test. Both groups averaged higher in the second study than in the first, but the between-group difference remained strong (Study II average scores for controls, 38.0 ± 1.4%; for inosine-treated rats, 67.1 ± 1.1%: Figure 1D; F = 227.8, P < 0.001). Between-group comparisons are based upon rats that met the inclusion criteria noted below.

In summary, inosine-treated rats performed markedly better than saline-treated controls on tests of general locomotion and sensorimotor integration after SCI. Rats performed equally well regardless of whether inosine was delivered i.v. or i.c.v., and the effects of *i.v.* inosine were reproducible across two independent studies.

### Inosine treatment does not affect lesion size

Histological analyses were carried out to investigate whether inosine affects secondary injury and to verify that we had completely transected the CST while leaving enough tissue intact to allow for the formation of detour circuits [29]. 

**Figure 2:**
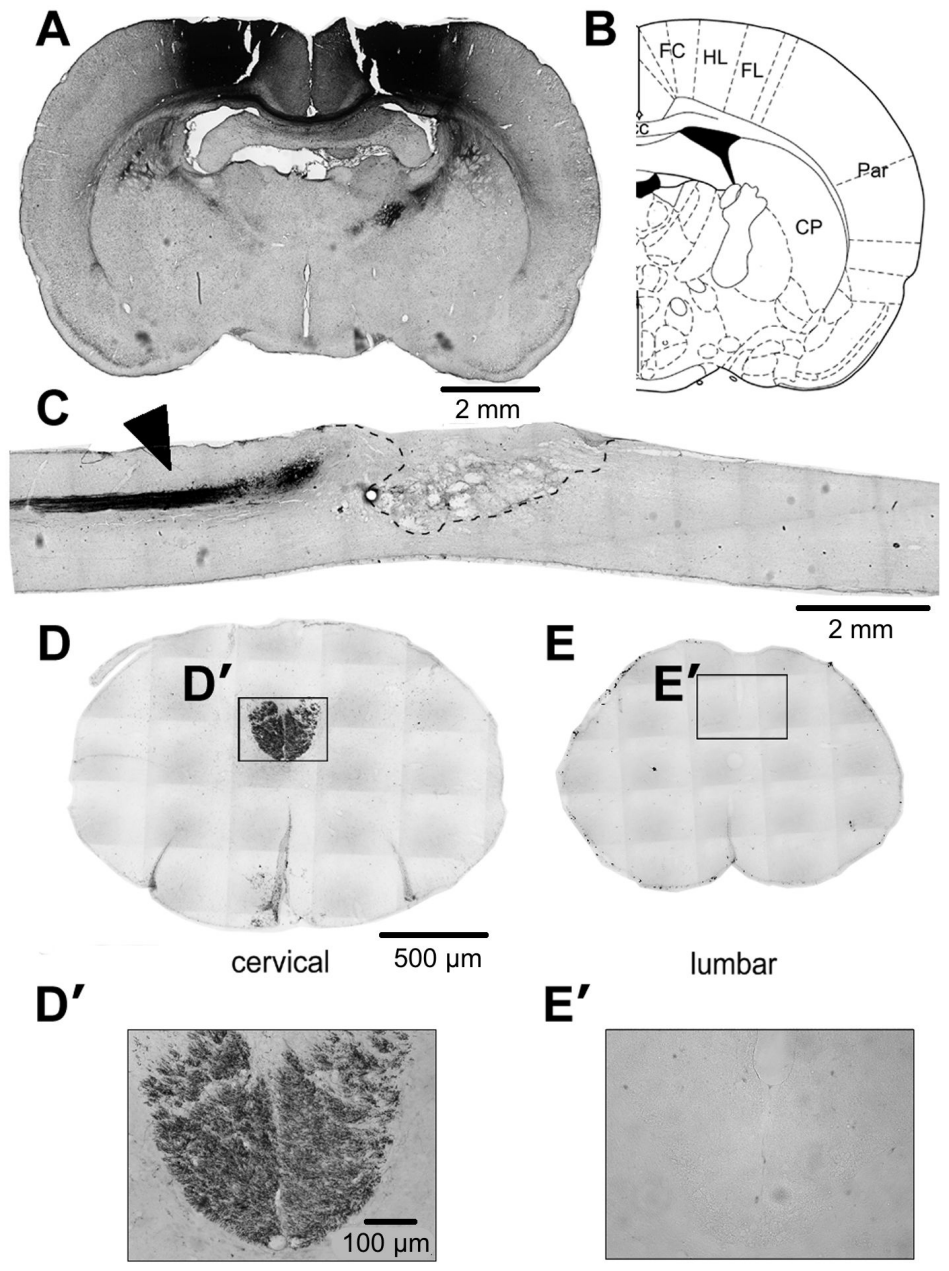
Interruption of the dorsal corticospinal tract (CST) after spinal cord injury (SCI). **A**. Hindlimb CST axons were labeled by injecting biotinylated dextran amine (BDA) into 9 sites in the hindlimb motor area of each hemisphere 4 weeks after SCI. Rats survived 2 more weeks to allow for BDA transport. **B**. Identity of brain structures (from the atlas of Paxinos and Watson, 1997). **C**. Parasagittal section through the thoracic cord shows labeled CST axons (*arrowhead*) rostral to the lesion (*dashed lines*). Note the absence of labeled fibers distal to the injury. **D**, **E**. Transverse sections of the spinal cord at the cervical (**D**) and lumbar (**E**) enlargements. Note heavy labeling at the cervical level (**D**, **D**') but absence of labeling in the lumbar cord (**E**, **E**'). Scale bar, 500 µm.

**Figure 3:**
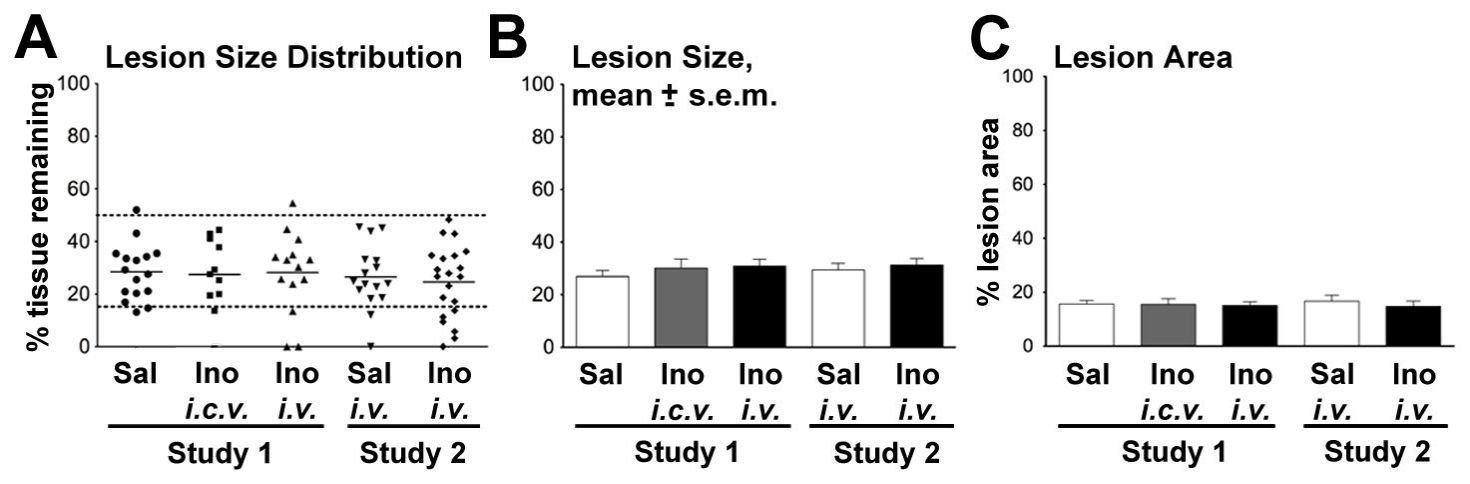
Inosine does not affect lesion size. **A**. Depth of lesions for all animals in Studies 1 and 2 (bars show group means; dashed lines show exclusion limits). **B**. lesion depth after excluding rats in which the lesion was either ≤ 50% or > 85% the depth of the spinal cord. **C**. Area of lesions (percentage of tissue lost within an 8 mm segment of spinal cord centered at the lesion epicenter). Inosine and saline-treated groups did not differ from each other in lesion depth or area (One-way ANOVA, Dunnet *a* *priori* test).

**Figure 4:**
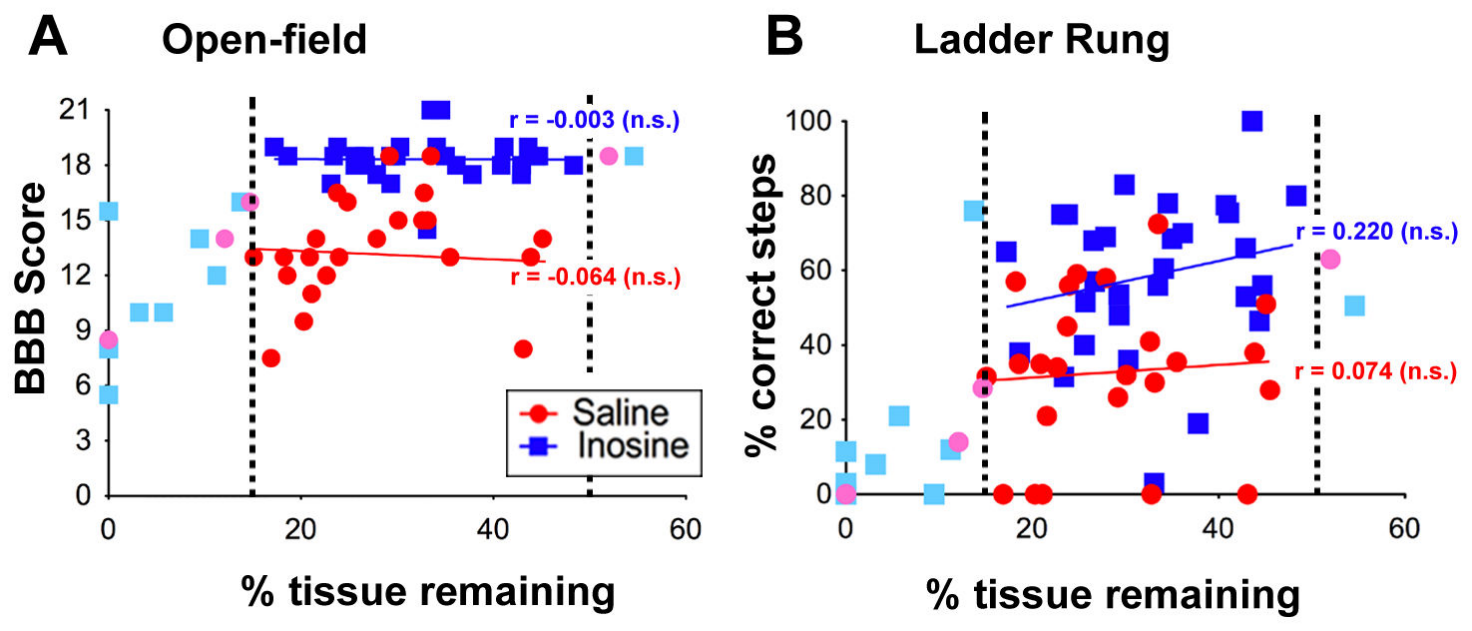
Behavioral outcome depends upon treatment but not depth of lesion. Scattergrams show open field behavior (**A**) and ladder rung scores (**B**) as a function of lesion depth. *Blue* *dots* represent rats treated with either i.c.v. or i.v. inosine, and *red* *dots* are rats treated with saline; dotted lines show the upper and lower bounds of our exclusion criteria. Within these bounds, inosine-treated rats out-performed controls, but there is no significant correlation (n.s.) between performance and lesion size. Performance on both tests falls off for lesions outside the lower limit for inclusion in the study.

Because even a small percentage of CST fibers can mediate skilled behavior [30], it was essential to verify that this tract was fully severed. This was accomplished by (a) visualizing the loss of the CST during surgery; (b) verifying the complete interruption of the CST in parasagittal sections through the lesion site; (c) verifying the absence of BDA-positive axons in coronal sections through the lumbar cord; and (d) excluding rats whose lesions were < 50% the depth of the spinal cord (Figure 2C-E). There were no significant between-group differences in the percentage of rats excluded based on these criteria (Figure 3A: 1 out of 28 saline-treated rats vs. 1 out of 39 inosine-treated rats: _*X*_^2^ = 0.07, P ~ 0.8). Rats were also excluded if lesions were ≥ 85% the depth of the spinal cord based on earlier studies showing that transection of ≥ 90% of the spinal cord prevents recovery of open-field behavior [29]. Our data show a marked drop-off in both open-field behavior and the horizontal ladder rung walking test when lesions exceeded 85% the depth of the cord despite inosine treatment (Figure 4). There were no significant between-group differences in the number of rats excluded using this second criterion (3 out of 28 saline-treated rats vs. 9 out of 39 inosine-treated rats: _*X*_^2^ = 1.12, P ~ 0.3; Figure 3A). To investigate possible between-group differences in numbers of rats excluded in the two groups further, we carried out a Mann-Whitney rank sum test (non-parametric) of the lesion depths of all animals and again found no significant difference between the inosine- and saline-treated groups (P = 0.84). In light of the appreciable motor function seen even in rats with the greatest lesions (Figure 4), it is possible that many of the apparently complete transections actually reflected tissue damage during sectioning or histological processing. A Mann-Whitney rank sum test of lesion depth vs. behavioral data that includes all rats, including those that that fell outside of our lesion criteria, continued to show highly significant differences between inosine- and saline-treated rats (BBB: P < 0.0001; Ladder-rung P < 0.0001). Animals remaining in the study after applying our exclusion criteria had lesions that were, on average, ~ 70% the depth of the cord regardless of treatment (Figure 3B). Saline and inosine-treated groups were also similar to each other in the cross-sectional areas of the lesions (Figure 3C). Together, these results indicate that (a) inosine treatment did not affect lesion size, and that (b) inter-group differences in performance are not a consequence of differences in lesion size. After applying our exclusion criteria, although there were clear inter-group differences on both tests, there were no significant within-group correlations between lesion depth and test performance (Figure 4). 

### Inosine-treated rats show increased CST sprouting and synaptic contacts

**Figure 5:**
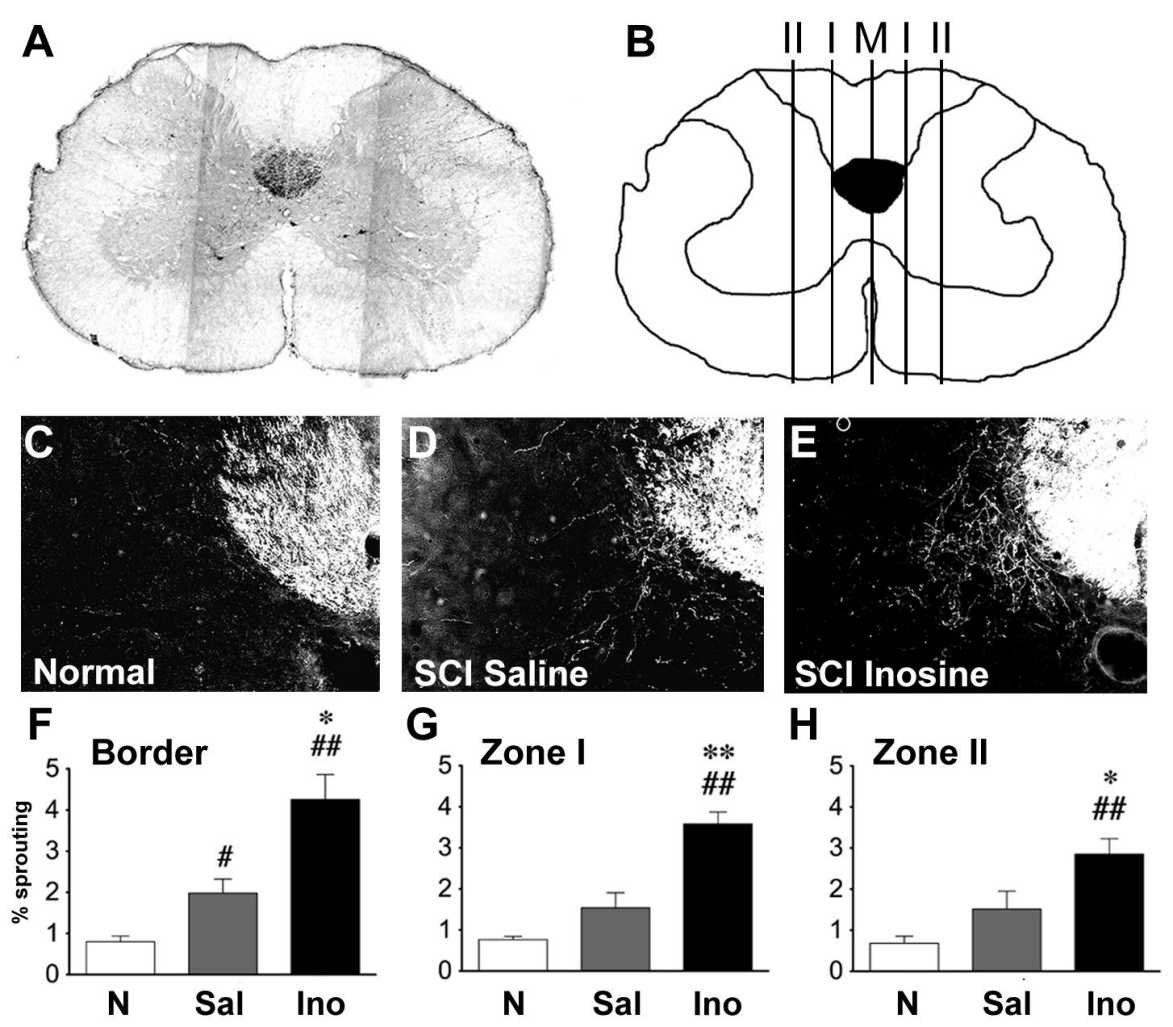
Inosine increases CST axon branching in the cervical spinal cord. **A**. Transverse section through the cervical enlargement. **B**. BDA-labeled CST collaterals were quantified at the border of the main (dorsomedial) CST (**B**), the midline (M), and at distances I and II from the midline. **C** - **E**, SCI alone increases CST sprouting in the gray matter compared to a sham-treated controls (D vs. C), and inosine enhances it further (E). **F** - **H**, Quantitation of CST sprouting into the gray matter at the indicated loci. ***#***P < 0.05, ^##^P < 0.01 compared to sham-operated controls; *P < 0.05, ** P < 0.01 compared to saline-treated rats with SCI. Axon counts were normalized by the total number of axons counted in the main CST at the cervical level (Mann-Whitney test).

CST axons transected at the thoracic level sprout collateral branches that can form synapses onto long propriospinal interneurons (LPSNs) at the cervical level of the spinal cord, forming a “detour circuit” that can partially restore cortical control to the lumbar level in the case of incomplete SCI [9-11]. We investigated the sprouting of hindlimb CST axons at 4 loci within the cervical spinal cord as shown in Figure 5B: (a) immediately adjacent to the principal bundle of the CST, (b) at the midline (M), (c) at a distance from the midline equal to the width of the dorsomedial CST (Zone I), and (d) at twice the latter distance (Zone II). Axon counts were normalized by the total number of BDA-labeled axons in the dorsal CST for each animal. In normal rats (n = 6), BDA labeling of the hindlimb motor cortex resulted in fewer than 1% of all labeled axons in the dorsal CST extending into the gray matter at the cervical level. Inosine- and saline-treated rats were selected for detailed histological analysis on a random basis and were coded so the person doing the analyses was unaware of the animals’ treatment. Several cases were rejected based on inadequate fixation or inadequate labeling of the CST.

CST transection and inosine treatment both increased axon sprouting. In the absence of additional treatments, dorsal hemisections of the thoracic spinal cord (n=5) increased the number of hindlimb axons that extended into the cervical gray matter two-fold (Figure 5F, P < 0.05; n.s. in G and H). Over and above this change, rats receiving inosine (n = 7) showed twice as many CST collaterals as saline-treated controls (Figure 5F - H: all P ≤ 0.05). This increase was highest adjacent to the dorsomedial CST, where the number of BDA-labeled axon branches was ~ 5-fold higher than in intact animals, and decreased at greater distances from the CST. Few CST fibers crossed the midline in any group, though there was a small increase after SCI (saline-treated compared to normal: P ≤ 0.05) and a further increase with inosine treatment (P ≤ 0.01, *not shown*).

**Figure 6:**
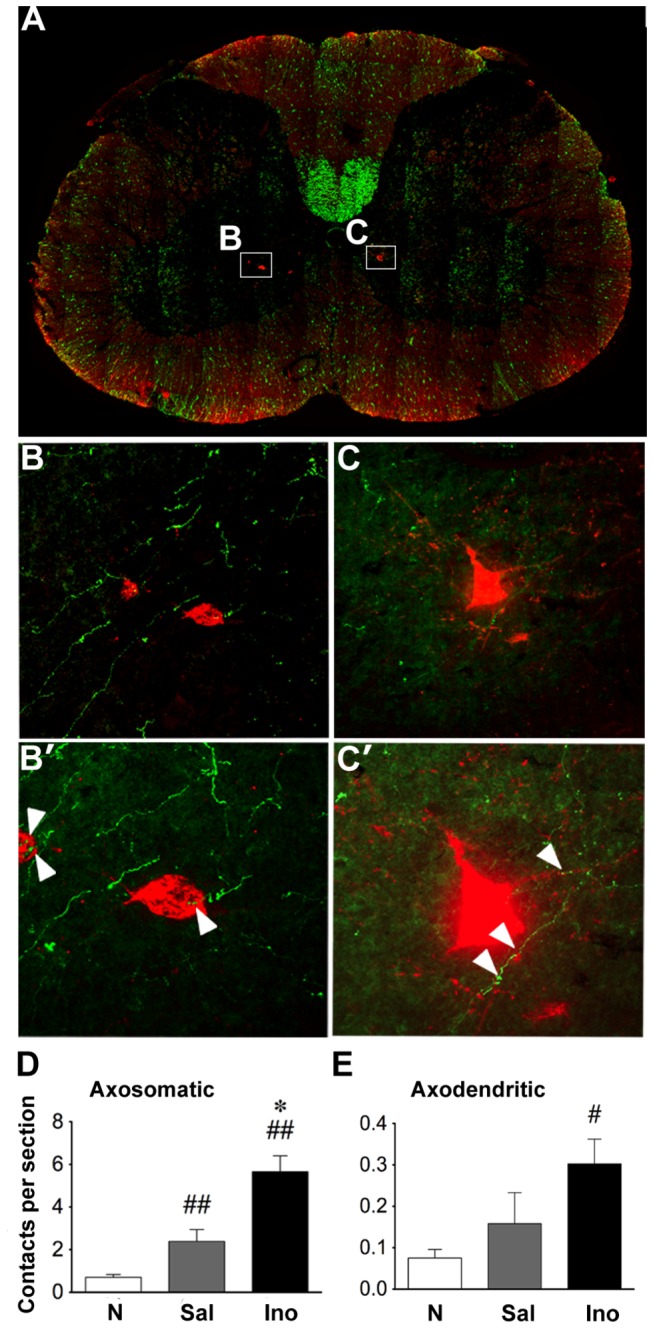
Inosine increases CST contacts onto LPSNs. **A**. Transverse section of an inosine-treated animal though the cervical enlargement shows position of LPSNs (*boxes*). **B**, **C**. Enlargement of areas in (**A**) show LPSNs (*red*) that project to the lumbar enlargement. **B**', **C**'. Higher magnification of cells in B and C. *Arrowheads* show BDA-labeled CST collaterals (*green*) contacting an LPSN soma (**B**') and proximal dendrites (**C**'). **D**, **E**, Quantitation of CST synapses upon LPSN somata and within 20 µm of labeled dendrites. SCI alone increases axosomatic synapses compared to sham-operated controls, and inosine increases the number of these to a greater extent (**D**). Inosine also increases putative axodendritic contacts compared to normal animals (**E**). *P < 0.05 compared to saline treatment, ^#^P < 0.05, ^##^P < 0.01 compared to sham-operated controls (Mann-Whitney).

We next investigated whether hindlimb CST axon collaterals contact LPSNs. Following the methods of Bareyre et. al. [9], we injected cholera toxin B fragment (CTB) into the lumbar enlargement to retrogradely label LPSNs that project from the cervical level of the spinal cord to the lumbar level, i.e., the level of hindlimb control. We then counted bouton-like swellings made by BDA-labeled CST axons onto CTB-labeled somata or within 20 µm of their visible dendrites (Figure 6). As before, results were normalized by the total number of BDA-positive axons in the CST to account for inter-animal variability in the overall extent of labeling. Axosomatic contacts were detected far more frequently than axodendric contacts, most likely because CTB labeling does not extend far into the dendritic arbor of LPSNs (Figure 6) and because, even if dendritic arbors were labeled, they may extend outside the plane of section. Rats with SCI and saline treatment had 3 times as many BDA-positive boutons that contacted CTB-positive somata as normal controls (P < 0.01). The inosine-treated group had twice as many of these contacts as saline-treated controls (P < 0.01, Figure 6D) and a qualitatively similar, though not statistically significant, increase in boutons within 20 µm of CTB+ dendrites (Figure 6E). Compared to normal rats, rats receiving inosine after SCI had ~ 10-fold more contacts of CST collaterals onto LPSN somata (Figure 6D, P ~ 0.001) and 4-fold more putative axodendritic contacts (Figure 6E, P < 0.05). 

Conceivably, the increase in BDA-labeled axons contacting CTB-labeled somata or dendrites noted above could reflect increases in the number of LPSNs that get labeled in the cervical enlargement following CTB injections in the lumbar area. Inosine-treated rats showed ~ 15% more labeled LPSNs following lumbar CTB injections than saline-treated controls, but this difference did not achieve statistical significance and is too small to account for the observed 3-fold difference in numbers of contacts (Normal, mean ± SEM = 50.7 ± 2.6; Saline, 42.8 ± 1.2; Inosine, 49.1 ± 1.8; differences not significant, one-way ANOVA). These results also indicate that, for rats that met our inclusion criteria, lesions did not greatly disrupt LPSN projections that course through the ventrolateral funiculus [31] and serve as the likely substrate for functional recovery [9].

**Figure 7:**
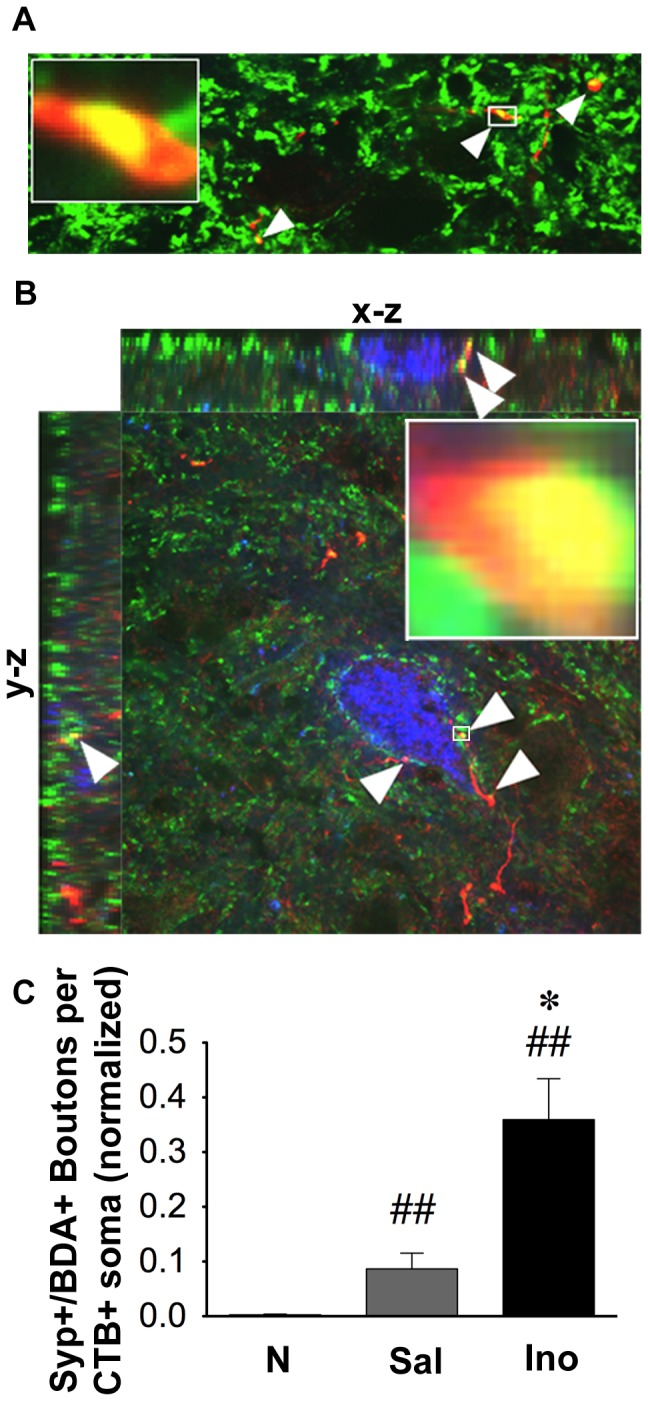
Inosine increases synaptic contacts between CST collaterals and LPSNs. **A**. Spinning-disc confocal image shows BDA-labeled boutons (*red*) in the cervical enlargement that arise from CST axon collaterals and express the presynaptic protein synaptophysin (*green*). **B**. Confocal image shows CST boutons co-localized with synaptophysin (*green*)-positive contacts upon a CTB-labeled LPSN (*blue*). Side images show co-localization in the x-z and y-z planes. **C**. Quantitation of synaptophysin- and BDA-positive boutons on LPSN somata. SCI alone increases synaptic contacts on LPSNs, and inosine causes a further 4-fold increase. Data are normalized by the total number of labeled axons in the main bundle of the CST. *P < 0.05 compared to saline treatment, ^##^P < 0.01 compared to normal control (Mann-Whitney test).

We next examined whether BDA-labeled boutons that contact LPSNs express synaptophysin and are therefore likely to represent physiological synapses. Normal rats showed almost no synaptophysin-positive CST contacts onto LPSNs (Figure 7C), whereas rats with SCI treated with saline showed significantly more (P < 0.01). Compared to the latter group, rats treated with inosine after SCI had nearly 4 times as many synaptophysin-positive boutons on CTB-positive LPSNs (Figure 7, P < 0.05). 

### Restoration of serotonergic innervation in the lumbar spinal cord

**Figure 8:**
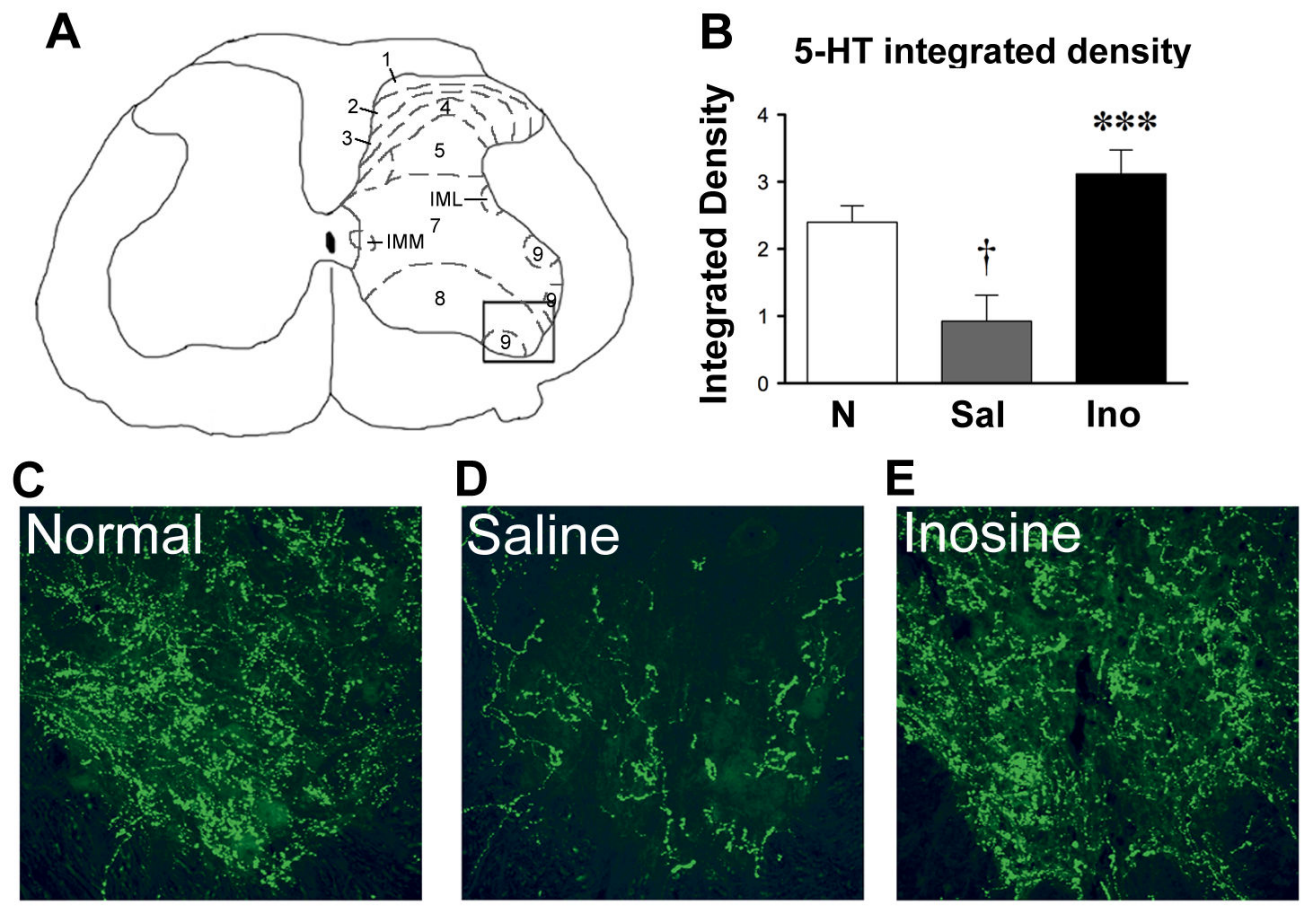
Inosine increases serotonergic input to the lumbar spinal cord. **A**. Schematic diagram of the lumbar enlargement. Box delineates area of 5-HT analysis in the ventral horn. **B**. Integrated intensity of 5-HT immunofluorescence. SCI decreases 5-HT immunofluorescence compared to normal controls. *i.v.* inosine restores 5-HT levels back to normal (F = 10.93; **P < 0.01 compared to saline treatment, ^†^P < 0.05 compared to sham-operated controls: One-way ANOVA, Dunnet *a* *priori* test). **C** - **E**. Photomicrographs show serotonergic fibers in the lumbar enlargement of sham-operated rats (**C**) and after SCI in rats treated with *i.v.* saline (**D**) or *i.v.* inosine (**E**).

Quantitation of 5HT immunostaining in the ventral horns of the lumbar spinal cord showed a 60% decline in levels of serotonin immunoreactivity following dorsal hemisections (n = 12). Inosine-treated rats (n = 14) exhibited levels of serotonin immunoreactivity comparable to those of normal rats (Figure 8). All cases used in this analysis had lesions depths that fell within the criteria described above.

### Dose-response study

**Figure 9:**
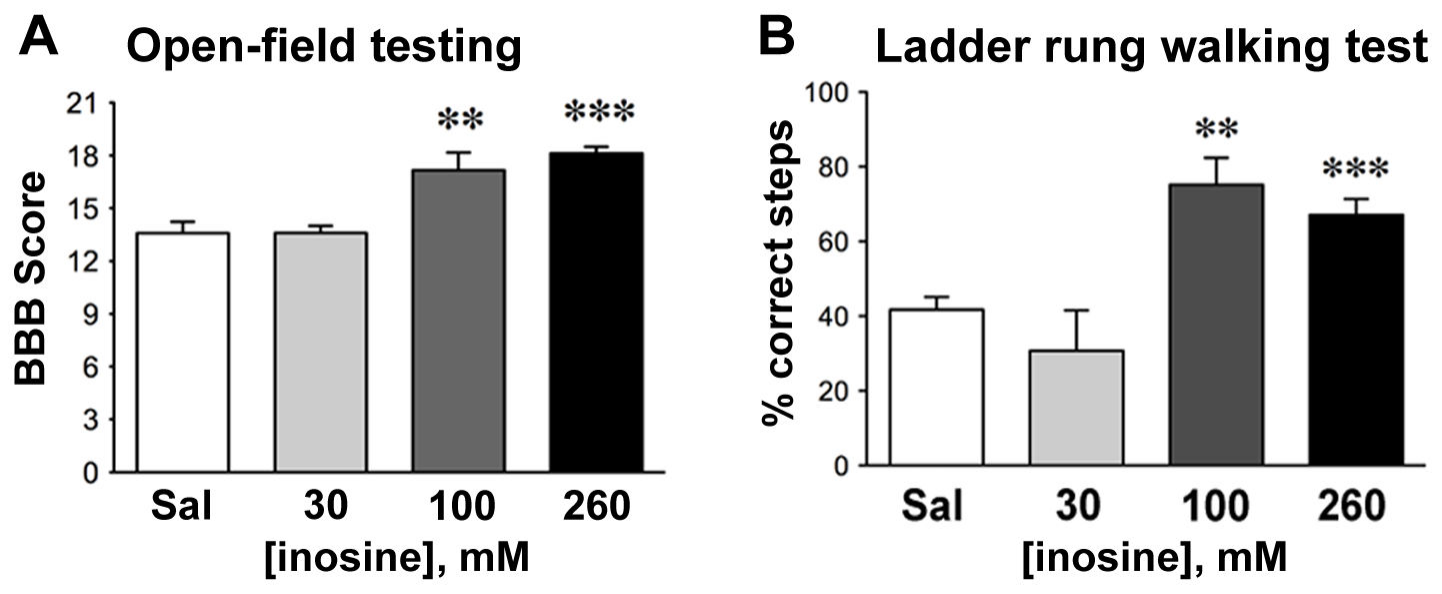
Dose-response relationship for intravenous inosine. **A**, **B**. Behavioral results 4 weeks after SCI. The effects of inosine reach a plateau at 100 mM in both the open-field (**A**) and ladder rung walking (**B**) tests. **C**, **D**. Behavioral test results over the 4 week test period. *P < 0.01; **P < 0.01; ***P < 0.01 (One-way ANOVA, Dunnet *a* *priori* test (a, b); two-way ANOVA, Bonferroni *a* *priori* test (c, d)).

A dose-response study demonstrated high levels of recovery in rats treated with 60% less inosine than in the above studies. In addition to the dosage tested above (260 mM), we tested *i.v.* inosine at nominal concentrations of 30 mM (n = 5) and 100 mM (n = 6). For statistical robustness, we included in the analysis rats that received 0 or 260 mM i.v. inosine in Studies 1 and 2 (total n = 17 for saline and n = 16 for 260 mM inosine). At 30 mM, inosine treatment did not improve outcome on either the BBB scale or the horizontal ladder rung-walking test. Although rats treated with 100 mM inosine showed a slow initial rate of recovery, their scores on the BBB scale and ladder-walk after 4 weeks were similar to those of rats receiving 260 mM inosine (P < 0.01 compared to saline-treated controls, both tests: Figure 9). 

## Discussion

Although most neural pathways cannot regenerate long distances after SCI in mature mammals, both injured and uninjured axons are able to extend collateral branches and form compensatory circuits that help improve outcome after incomplete spinal lesions [9,12,32-35]. Clinically, such sprouting is thought to contribute to the partial recovery that is often seen in patients with incomplete lesions [36]. In the present study, rats treated with inosine showed considerably more axon sprouting and more corticospinal-LPSN synapses after incomplete SCI than saline-treated rats, along with superior control of hindlimb function. 

The corticospinal tract (CST) mediates skilled movements of the distal extremities and has been studied extensively due to its importance for voluntary control in humans [37]. In mice and rats, CST damage has only a transitory effect on general locomotion but severely impairs skilled use of the limbs [23]. Even without special treatments, CST axons sprout collateral branches after SCI that can form novel circuits, and this process can be augmented by physiological activity, trophic factors, or counteracting cell-extrinsic inhibitors of axon growth [11,38-45]. Following CST injury at the mid-thoracic level, severed axons arising from pyramidal cells in the hindlimb motor cortex sprout collateral branches rostral to the injury site that synapse onto long propriospinal interneurons in laminae V-VII of the cervical cord, forming “detour circuits” that partially restore cortical control to the hindlimbs [9-11,46]. In conformity with these studies, we observed a two-fold increase in CST sprouting after dorsal hemisections in saline-treated controls. Inosine-treated animals showed twice as much axon sprouting as saline-treated controls and 4 times the number of synaptic contacts between CST collaterals and long propriospinal interneurons (LPSNs) that project from the cervical enlargement to the lumbar level. The role of this circuit in functional recovery has been demonstrated by trans-synaptic labeling and electrophysiology [9,44]. We observed many more axosomatic than axodendritic contacts, which may be a consequence of the failure of the retrograde tracer (CTB) to fill distal portions of dendrites, or may be characteristic of these novel connections. At a functional level, although untreated controls showed partial recovery 4 weeks after SCI, the recovery in inosine-treated animals was much stronger. Compared to saline-treated controls, rats treated with inosine showed much greater accuracy in hindlimb placement in the ladder-rung walking test, a measure of cortical control of the distal extremities [23], and a 4-5 point difference on the BBB scale. Unlike the CST, LPSNs can regenerate axons when transected [47]. However, our retrograde labeling studies suggest that inosine did not greatly alter the projections of these neurons. 

Because a small residue of CST fibers can mediate appreciable skilled hindlimb movements [29], we excluded rats whose lesions spared axons in the dorsal CST. We also excluded rats in which lesions were > 85% the depth of the cord, as such lesions would damage the ventrolateral funiculus [31] that carries propriospinal axons that are important for the recovery of skilled hindlimb control [9]. Our behavioral analysis confirmed the inability of rats to recover skilled ladder rung-walking after such extensive lesions. Following the exclusion of animals based on these criteria, inosine- and saline-treated groups were closely matched for lesion size and area, and the observed differences in behavior can therefore not be attributed to differences in tissue sparing. In conformity with these results, several prior studies have reported beneficial effects of inosine after stroke or traumatic brain injury in the absence of neuroprotection [20,21,27]. However, one study reported inosine to be neuroprotective when administered prior to SCI [48]. 

Descending serotonergic projections originate in the medullary raphe nuclei, course through the ventrolateral and ventral funiculi, and terminate in the ventral horn and other parts of the spinal gray matter [49]. The raphespinal pathway contributes to maintaining posture, initiating movement, and modulating the central pattern generator (Jordan et al., 2008), and is critical for the recovery of general locomotion after SCI [44,50-54]. Recovery of locomotion after SCI can also be mediated in part by constitutively active 5HT2 receptors when raphespinal input is eliminated [54]. As expected, our lesions strongly reduced serotonergic input to the ventral gray matter of the lumbar spinal cord [6,44,55], but inosine treatment restored 5-HT immunoreactivity to normal levels. These rats displayed greater forelimb-hindlimb coordination and limb placement than saline-treated controls, returning to near-normal levels of performance after 4 weeks. However, because the recovery of locomotion depends upon multiple systems that course through the ventral spinal cord [23], and because serotonergic receptors can be constitutively active, the extent to which the improvements in locomotion seen here depended upon raphespinal sprouting *per se* is uncertain. Raphespinal sprouting has also been linked to improvements in locomotion after counteracting inhibitory proteins associated with myelin [6,44,55]. 

Inosine is a naturally occurring metabolite of adenosine that can diffuse across the neuronal cell membrane and activate Mst3b, a protein kinase that is part of a cell-signaling pathway that controls axon outgrowth [13,14]. Low micromolar concentrations of inosine induce axon outgrowth from embryonic cortical neurons and other neural populations in culture [13,15,16,20], and *in vivo*, inosine stimulates axon sprouting from CST axons originating in the undamaged hemisphere after a unilateral stroke or traumatic brain injury [18,20,21,27]. Inosine was also reported to promote CST sprouting after unilateral transection of the contralateral CST [56], although this latter finding was not replicated in another study [57], possibly due to important methodological differences [58]. 

Besides its effects on axon sprouting, inosine has been reported to suppress the response of cortical neurons to glutamate [59], enhance inhibition via benzodiazepine receptors [60], limit the production of inflammatory cytokines [61,62], and attenuate hypoxia-induced astrocyte death [63,64]. Uric acid, a primary metabolite of inosine, prevents peroxynitrite-induced protein damage, protects the blood-brain barrier, and has potent anti-inflammatory effects [65,66]. Importantly, inosine has a history of safe use in humans. It is used in cardiac patients in several countries [67] and is often taken by athletes, though with questionable benefit [68]. Based upon the effectiveness of the inosine metabolite uric acid as an antioxidant, clinical trials are underway using oral inosine in Parkinson’s Disease http://clinicaltrials.gov/ct2/show/NCT00833690 and multiple sclerosis http://clinicaltrials.gov/ct2/show/NCT00067327. 

We found inosine to reach maximal effectiveness at ~ 40 mg/da/kg *i.v*. This is somewhat higher than the dose commonly administered in China *i.v.* for multiple indications (4-20 mg/da/kg: Dr. Gong Ju, personal communication) but lower than the recommended oral dose as a health supplement (~150 mg/kg/da). Inosine is effective at a much lower dose when delivered directly into the brain through an indwelling ventricular catheter, but this route is clinically undesirable due to risk of cerebral infection. The efficacy of *i.v.* administration is consistent with earlier studies showing appreciable permeability through the blood-brain-barrier [69,70]. 

Although this study has demonstrated inosine’s efficacy in a dorsal spinal cord hemisection model, transection of defined fiber tracts seldom occurs clinically, and a contusion model of injury would be valuable to further assess the value of inosine in a clinical setting. In addition, although our results show an effect of inosine on the formation of novel CST-LPSN contacts, it is likely that other pathways also contribute to the observed functional improvements. Among the pathways damaged by a dorsal hemisection is the rubrospinal tract, which is important for volitional control, though we have not examined changes in this pathway. In addition, our results show extensive sprouting of the raphespinal tract, which is important for general locomotion and right-left coordination. It might be noted, however, that the CST sprouting that we report here probably underestimates the formation of novel CST-LPSN synapses since we were only able to observe CST contacts onto LPSN somata and proximal dendrites. Possible contacts onto the distal dendritic arbor were not visualized due to the limited filling of cell bodies by retrogradely transported CTB. 

Because of its demonstrated efficacy in multiple animal models of neurological injury (SCI, stroke, traumatic brain injury), its clinical safety in humans, and the feasibility of *i.v.* delivery, inosine would appear to be a good candidate to advance towards clinical trials for treating patients with SCI, perhaps in combination with complementary treatments. Inosine delivered *i.c.v.* potentiates the effects of environmental enrichment, training, and a Nogo receptor antagonist after unilateral cortical damage [20,27], and it may similarly augment the effects of these and other treatments that improve outcome after SCI such as physiological activity or stem cell implants [6,8]. 

## References

[1] Filbin MT (2003) Myelin-associated inhibitors of axonal regeneration in the adult mammalian CNS. Nat Rev Neurosci 4: 703-713. doi:10.1038/nrn1195. PubMed: 12951563. 12951563

[2] Fitch MT, Silver J (2008) CNS injury, glial scars, and inflammation: Inhibitory extracellular matrices and regeneration failure. Exp Neurol 209: 294-301. doi:10.1016/j.expneurol.2007.05.014. PubMed: 17617407. 17617407 PMC2268907

[3] Rossignol S, Schwab M, Schwartz M, Fehlings MG (2007) Spinal cord injury: time to move? J Neurosci 27: 11782-11792. doi:10.1523/JNEUROSCI.3444-07.2007. PubMed: 17978014. 17978014 PMC6673354

[4] Liu K, Tedeschi A, Park KK, He Z (2011) Neuronal intrinsic mechanisms of axon regeneration. Annu Rev Neurosci 34: 131-152. doi:10.1146/annurev-neuro-061010-113723. PubMed: 21438684. 21438684

[5] Schwab ME (2002) Repairing the injured spinal cord. Science 295: 1029-1031. doi:10.1126/science.1067840. PubMed: 11834824. 11834824

[6] van den Brand R, Heutschi J, Barraud Q, DiGiovanna J, Bartholdi K et al. (2012) Restoring voluntary control of locomotion after paralyzing spinal cord injury. Science 336: 1182-1185. doi:10.1126/science.1217416. PubMed: 22654062. 22654062

[7] Liu K, Lu Y, Lee JK, Samara R, Willenberg R et al. (2010) PTEN deletion enhances the regenerative ability of adult corticospinal neurons. Nat Neurosci 13: 1075-1081. doi:10.1038/nn.2603. PubMed: 20694004. 20694004 PMC2928871

[8] Lu P, Wang Y, Graham L, McHale K, Gao M et al. (2012) Long-distance growth and connectivity of neural stem cells after severe spinal cord injury. Cell 150: 1264-1273. doi:10.1016/j.cell.2012.08.020. PubMed: 22980985. 22980985 PMC3445432

[9] Bareyre FM, Kerschensteiner M, Raineteau O, Mettenleiter TC, Weinmann O et al. (2004) The injured spinal cord spontaneously forms a new intraspinal circuit in adult rats. Nat Neurosci 7: 269-277. doi:10.1038/nn1195. PubMed: 14966523. 14966523

[10] Lang C, Guo X, Kerschensteiner M, Bareyre FM (2012) Single Collateral Reconstructions Reveal Distinct Phases of Corticospinal Remodeling after Spinal Cord. Injury - PLOS ONE 7: 30411 30411. doi:10.1371/journal.pone.0030461. PMC3265484 22291960

[11] Vavrek R, Girgis J, Tetzlaff W, Hiebert GW, Fouad K (2006) BDNF promotes connections of corticospinal neurons onto spared descending interneurons in spinal cord injured rats. Brain 129: 1534-1545. doi:10.1093/brain/awl087. PubMed: 16632552. 16632552

[12] Weidner N, Ner A, Salimi N, Tuszynski MH (2001) Spontaneous corticospinal axonal plasticity and functional recovery after adult central nervous system injury. Proc Natl Acad Sci U S A 98: 3513-3518. doi:10.1073/pnas.051626798. PubMed: 11248109. 11248109 PMC30684

[13] Irwin N, Li YM, O'Toole JE, Benowitz LI (2006) Mst3b, a purine-sensitive Ste20-like protein kinase, regulates axon outgrowth. Proc Natl Acad Sci U S A 103: 18320-18325. doi:10.1073/pnas.0605135103. PubMed: 17114295. 17114295 PMC1838749

[14] Lorber B, Howe ML, Benowitz LI, Irwin N (2009) Mst3b, an Ste20-like kinase, regulates axon regeneration in mature CNS and PNS pathways. Nat Neurosci 12: 1407-1414. doi:10.1038/nn.2414. PubMed: 19855390. 19855390 PMC2770175

[15] Zurn AD, Do KQ (1988) Purine metabolite inosine is an adrenergic neurotrophic substance for cultured chicken sympathetic neurons. Proc Natl Acad Sci U S A 85: 8301-8305. doi:10.1073/pnas.85.21.8301. PubMed: 3186724. 3186724 PMC282417

[16] Benowitz LI, Jing Y, Tabibiazar R, Jo SA, Petrausch B et al. (1998) Axon outgrowth is regulated by an intracellular purine-sensitive mechanism in retinal ganglion cells. J Biol Chem 273: 29626-29634. doi:10.1074/jbc.273.45.29626. PubMed: 9792672. 9792672

[17] Petrausch B, Tabibiazar R, Roser T, Jing Y, Goldman D et al. (2000) A purine-sensitive pathway regulates multiple genes involved in axon regeneration in goldfish retinal ganglion cells. J Neurosci 20: 8031-8041. PubMed: 11050124. 11050124 10.1523/JNEUROSCI.20-21-08031.2000 PMC6772744

[18] Chen P, Goldberg DE, Kolb B, Lanser M, Benowitz LI (2002) Inosine induces axonal rewiring and improves behavioral outcome after stroke. Proc Natl Acad Sci U S A 99: 9031-9036. doi:10.1073/pnas.132076299. PubMed: 12084941. 12084941 PMC124418

[19] Smith JM, Lunga P, Story D, Harris N, Belle JL et al. (2007) Inosine promotes recovery of skilled motor function in a model of focal brain injury. Brain 130: 915-925. PubMed: 17293357. 17293357 10.1093/brain/awl393

[20] Zai L, Ferrari C, Dice C, Subbaiah S, Havton LA et al. (2011) Inosine augments the effects of a Nogo receptor blocker and of environmental enrichment to restore skilled forelimb use after stroke. J Neurosci 31: 5977-5988. doi:10.1523/JNEUROSCI.4498-10.2011. PubMed: 21508223. 21508223 PMC3101108

[21] Zai L, Ferrari C, Subbaiah S, Havton LA, Coppola G et al. (2009) Inosine alters gene expression and axonal projections in neurons contralateral to a cortical infarct and improves skilled use of the impaired limb. J Neurosci 29: 8187-8197. doi:10.1523/JNEUROSCI.0414-09.2009. PubMed: 19553458. 19553458 PMC2856695

[22] Basso DM, Beattie MS, Bresnahan JC (1995) A sensitive and reliable locomotor rating scale for open field testing in rats. J Neurotrauma 12: 1-21. doi:10.1089/neu.1995.12.1. PubMed: 7783230. 7783230

[23] Metz GA, Whishaw IQ (2002) Cortical and subcortical lesions impair skilled walking in the ladder rung walking test: a new task to evaluate fore- and hindlimb stepping, placing, and co-ordination. J Neurosci Methods 115: 169-179. doi:10.1016/S0165-0270(02)00012-2. PubMed: 11992668. 11992668

[24] Paxinos G, Watson C (1986) The Rat Brain in Stereotaxic Coordinates. Boston: Academic Press, Inc.

[25] Fonoff ET, Pereira JF Jr., Camargo LV, Dale CS, Pagano RL et al. (2009) Functional mapping of the motor cortex of the rat using transdural electrical stimulation. Behav Brain Res 202: 138-141. doi:10.1016/j.bbr.2009.03.018. PubMed: 19447290. 19447290

[26] Havton L, Kellerth JO (1987) Regeneration by supernumerary axons with synaptic terminals in spinal motoneurons of cats. Nature 325: 711-714. doi:10.1038/325711a0. PubMed: 3821862. 3821862

[27] Smith JM, Lunga P, Story D, Harris N, Le Belle J et al. (2007) Inosine promotes recovery of skilled motor function in a model of focal brain injury. Brain 130: 915-925. PubMed: 17293357. 17293357 10.1093/brain/awl393

[28] McEwen ML, Springer JE (2006) Quantification of locomotor recovery following spinal cord contusion in adult rats. J Neurotrauma 23: 1632-1653. doi:10.1089/neu.2006.23.1632. PubMed: 17115910. 17115910

[29] Schucht P, Raineteau O, Schwab ME, Fouad K (2002) Anatomical correlates of locomotor recovery following dorsal and ventral lesions of the rat spinal cord. Exp Neurol 176: 143-153. doi:10.1006/exnr.2002.7909. PubMed: 12093091. 12093091

[30] Steward O, Zheng B, Tessier-Lavigne M (2003) False resurrections: distinguishing regenerated from spared axons in the injured central nervous system. J Comp Neurol 459: 1-8. doi:10.1002/cne.10593. PubMed: 12629662. 12629662

[31] Reed WR, Shum-Siu A, Onifer SM, Magnuson DS (2006) Inter-enlargement pathways in the ventrolateral funiculus of the adult rat spinal cord. Neuroscience 142: 1195-1207. doi:10.1016/j.neuroscience.2006.07.017. PubMed: 16938403. 16938403 PMC3741649

[32] Cafferty WB, McGee AW, Strittmatter SM (2008) Axonal growth therapeutics: regeneration or sprouting or plasticity? Trends Neurosci 31: 215-220. doi:10.1016/j.tins.2008.02.004. PubMed: 18395807. 18395807 PMC2678051

[33] Onifer SM, Smith GM, Fouad K (2011) Plasticity after spinal cord injury: relevance to recovery and approaches to facilitate it. Neurotherapeutics 8: 283-293. doi:10.1007/s13311-011-0034-4. PubMed: 21384221. 21384221 PMC3101826

[34] Rosenzweig ES, Courtine G, Jindrich DL, Brock JH, Ferguson AR et al. (2010) Extensive spontaneous plasticity of corticospinal projections after primate spinal cord injury. Nat Neurosci 13: 1505-1510. doi:10.1038/nn.2691. PubMed: 21076427. 21076427 PMC3144760

[35] Darlot F, Cayetanot F, Gauthier P, Matarazzo V, Kastner A (2012) Extensive respiratory plasticity after cervical spinal cord injury in rats : Axonal sprouting and rerouting of ventrolateral bulbospinal pathways. Exp Neurol 236: 88-102. doi:10.1016/j.expneurol.2012.04.004. PubMed: 22542946. 22542946

[36] Rossignol S, Frigon A (2011) Recovery of locomotion after spinal cord injury: some facts and mechanisms. Annu Rev Neurosci 34: 413-440. doi:10.1146/annurev-neuro-061010-113746. PubMed: 21469957. 21469957

[37] Lemon RN (2008) Descending pathways in motor control. Annu Rev Neurosci 31: 195-218. doi:10.1146/annurev.neuro.31.060407.125547. PubMed: 18558853. 18558853

[38] Tuszynski MH, Grill R, Jones LL, Brant A, Blesch A et al. (2003) NT-3 gene delivery elicits growth of chronically injured corticospinal axons and modestly improves functional deficits after chronic scar resection. Exp Neurol 181: 47-56. doi:10.1016/S0014-4886(02)00055-9. PubMed: 12710933. 12710933

[39] Oertle T, van der Haar ME, Bandtlow CE, Robeva A, Burfeind P et al. (2003) Nogo-A inhibits neurite outgrowth and cell spreading with three discrete regions. J Neurosci 23: 5393-5406. PubMed: 12843238. 12843238 10.1523/JNEUROSCI.23-13-05393.2003 PMC6741224

[40] Schäbitz WR, Berger C, Kollmar R, Seitz M, Tanay E et al. (2004) Effect of brain-derived neurotrophic factor treatment and forced arm use on functional motor recovery after small cortical ischemia. Stroke 35: 992-997. doi:10.1161/01.STR.0000119754.85848.0D. PubMed: 14988579. 14988579

[41] Cafferty WB, Strittmatter SM (2006) The Nogo-Nogo receptor pathway limits a spectrum of adult CNS axonal growth. J Neurosci 26: 12242-12250. doi:10.1523/JNEUROSCI.3827-06.2006. PubMed: 17122049. 17122049 PMC2848954

[42] Maier IC, Baumann K, Thallmair M, Weinmann O, Scholl J et al. (2008) Constraint-induced movement therapy in the adult rat after unilateral corticospinal tract injury. J Neurosci 28: 9386-9403. doi:10.1523/JNEUROSCI.1697-08.2008. PubMed: 18799672. 18799672 PMC6671131

[43] Wang X, Budel S, Baughman K, Gould G, Song KH et al. (2009) Ibuprofen enhances recovery from spinal cord injury by limiting tissue loss and stimulating axonal growth. J Neurotrauma 26: 81-95. doi:10.1089/neu.2007.0464. PubMed: 19125588. 19125588 PMC2913782

[44] Courtine G, Gerasimenko Y, van den Brand R, Yew A, Musienko P et al. (2009) Transformation of nonfunctional spinal circuits into functional states after the loss of brain input. Nat Neurosci 12: 1333-1342. doi:10.1038/nn.2401. PubMed: 19767747. 19767747 PMC2828944

[45] Fang PC, Barbay S, Plautz EJ, Hoover E, Strittmatter SM et al. (2010) Combination of NEP 1-40 treatment and motor training enhances behavioral recovery after a focal cortical infarct in rats. Stroke 41: 544-549. doi:10.1161/STROKEAHA.110.588137. PubMed: 20075346. 20075346 PMC2853474

[46] Ballermann M, Fouad K (2006) Spontaneous locomotor recovery in spinal cord injured rats is accompanied by anatomical plasticity of reticulospinal fibers. Eur J Neurosci 23: 1988-1996. PubMed: 16630047. 16630047 10.1111/j.1460-9568.2006.04726.x

[47] Fenrich KK, Rose PK (2009) Spinal interneuron axons spontaneously regenerate after spinal cord injury in the adult feline. J Neurosci 29: 12145-12158. doi:10.1523/JNEUROSCI.0897-09.2009. PubMed: 19793972. 19793972 PMC6666142

[48] Liu F, You SW, Yao LP, Liu HL, Jiao XY et al. (2006) Secondary degeneration reduced by inosine after spinal cord injury in rats. Spinal Cord 44: 421-426. PubMed: 16317421. 16317421 10.1038/sj.sc.3101878

[49] Skagerberg G, Björklund A (1985) Topographic principles in the spinal projections of serotonergic and non-serotonergic brainstem neurons in the rat. Neuroscience 15: 445-480. doi:10.1016/0306-4522(85)90225-8. PubMed: 4022334. 4022334

[50] Barbeau H, Rossignol S (1994) Enhancement of locomotor recovery following spinal cord injury. Curr Opin Neurol 7: 517-524. doi:10.1097/00019052-199412000-00008. PubMed: 7866583. 7866583

[51] Ribotta MG, Provencher J, Feraboli-Lohnherr D, Rossignol S, Privat A et al. (2000) Activation of locomotion in adult chronic spinal rats is achieved by transplantation of embryonic raphe cells reinnervating a precise lumbar level. J Neurosci 20: 5144-5152. PubMed: 10864971. 10864971 10.1523/JNEUROSCI.20-13-05144.2000 PMC6772289

[52] Kim D, Adipudi V, Shibayama M, Giszter S, Tessler A et al. (1999) Direct agonists for serotonin receptors enhance locomotor function in rats that received neural transplants after neonatal spinal transection. J Neurosci 19: 6213-6224. PubMed: 10407057. 10407057 10.1523/JNEUROSCI.19-14-06213.1999 PMC6783084

[53] Saruhashi Y, Young W, Perkins R (1996) The recovery of 5-HT immunoreactivity in lumbosacral spinal cord and locomotor function after thoracic hemisection. Exp Neurol 139: 203-213. doi:10.1006/exnr.1996.0094. PubMed: 8654523. 8654523

[54] Fouad K, Rank MM, Vavrek R, Murray KC, Sanelli L et al. (2010) Locomotion after spinal cord injury depends on constitutive activity in serotonin receptors. J Neurophysiol 104: 2975-2984. doi:10.1152/jn.00499.2010. PubMed: 20861436. 20861436 PMC3007654

[55] Kim JE, Liu BP, Yang X, Strittmatter SM (2003) Recovery from spinal cord injury in mice lacking the Nogo-66 receptor. Program No 41511 Abstract Viewer and Itinerary Planner Washington, DC: Society for Neuroscience, 2003 CD-ROM

[56] Benowitz LI, Goldberg DE, Madsen JR, Soni D, Irwin N (1999) Inosine stimulates extensive axon collateral growth in the rat corticospinal tract after injury. Proc Natl Acad Sci U S A 96: 13486-13490. doi:10.1073/pnas.96.23.13486. PubMed: 10557347. 10557347 PMC23974

[57] Steward O, Sharp K, Yee KM (2012) A re-assessment of the effects of intracortical delivery of inosine on transmidline growth of corticospinal tract axons after unilateral lesions of the medullary pyramid. Exp Neurol 233: 662-673. PubMed: 21946267. 21946267 10.1016/j.expneurol.2011.09.019 PMC3652674

[58] Benowitz L (2012) Author's response to Steward et al., "A re-assessment of the effects of intra-cortical delivery of inosine....". Exp Neurol 233: 674-676. PubMed: 22001160. 22001160 10.1016/j.expneurol.2011.09.034

[59] Shen H, Chen GJ, Harvey BK, Bickford PC, Wang Y (2005) Inosine reduces ischemic brain injury in rats. Stroke 36: 654-659. doi:10.1161/01.STR.0000155747.15679.04. PubMed: 15692110. 15692110

[60] Marangos PJ, Trams E, Clark-Rosenberg RL, Paul SM, Skolnick P (1981) Anticonvulsant doses of inosine result in brain levels sufficient to inhibit [3H] diazepam binding. Psychopharmacology (Berl) 75: 175-178. doi:10.1007/BF00432183. 6275442

[61] Haskó G, Sitkovsky MV, Szabó C (2004) Immunomodulatory and neuroprotective effects of inosine. Trends Pharmacol Sci 25: 152-157. doi:10.1016/j.tips.2004.01.006. PubMed: 15019271. 15019271

[62] Haskó G, Kuhel DG, Németh ZH, Mabley JG, Stachlewitz RF et al. (2000) Inosine inhibits inflammatory cytokine production by a posttranscriptional mechanism and protects against endotoxin-induced shock. J Immunol 164: 1013-1019. PubMed: 10623851. 10623851 10.4049/jimmunol.164.2.1013

[63] Haun SE, Segeleon JE, Trapp VL, Clotz MA, Horrocks LA (1996) Inosine mediates the protective effect of adenosine in rat astrocyte cultures subjected to combined glucose-oxygen deprivation. J Neurochem 67: 2051-2059. PubMed: 8863513. 8863513 10.1046/j.1471-4159.1996.67052051.x

[64] Jurkowitz MS, Litsky ML, Browning MJ, Hohl CM (1998) Adenosine, inosine, and guanosine protect glial cells during glucose deprivation and mitochondrial inhibition: correlation between protection and ATP preservation. J Neurochem 71: 535-548. PubMed: 9681443. 9681443 10.1046/j.1471-4159.1998.71020535.x

[65] Scott GS, Spitsin SV, Kean RB, Mikheeva T, Koprowski H et al. (2002) Therapeutic intervention in experimental allergic encephalomyelitis by administration of uric acid precursors. Proc Natl Acad Sci U S A 99: 16303-16308. doi:10.1073/pnas.212645999. PubMed: 12451183. 12451183 PMC138606

[66] Scott GS, Cuzzocrea S, Genovese T, Koprowski H, Hooper DC (2005) Uric acid protects against secondary damage after spinal cord injury. Proc Natl Acad Sci U S A 102: 3483-3488. doi:10.1073/pnas.0500307102. PubMed: 15728348. 15728348 PMC552934

[67] Czarnecki W, Czarnecki A (1989) Haemodynamic effects of inosine. A new drug for failing human heart? Pharmacolpharmacol Res 21: 587-594. doi:10.1016/1043-6618(89)90200-4. 2594615

[68] Starling RD, Trappe TA, Short KR, Sheffield-Moore M, Jozsi AC et al. (1996) Effect of inosine supplementation on aerobic and anaerobic cycling performance. Med Sci Sports Exerc 28: 1193-1198. doi:10.1097/00005768-199609000-00017. PubMed: 8883009. 8883009

[69] Cornford EM, Oldendorf WH (1975) Independent blood-brain barrier transport systems for nucleic acid precursors. Biochim Biophys Acta 394: 211-219. doi:10.1016/0005-2736(75)90259-X. PubMed: 1138930. 1138930

[70] Nakagawa S, Guroff G (1973) The uptake of purines by rat brain in vivo and in vitro. J Neurochem 20: 1141-1149. PubMed: 4697876. 4697876 10.1111/j.1471-4159.1973.tb00084.x

